# Graves’ Ophthalmopathy in the Setting of Primary Hypothyroidism

**DOI:** 10.7759/cureus.24954

**Published:** 2022-05-12

**Authors:** Sarah Alajmi, Sara Alshehri, Aishah Ekhzaimy

**Affiliations:** 1 Department of Internal Medicine, King Saud University Medical City, Riyadh, SAU

**Keywords:** thyroid-associated ophthalmopathy, hashimoto’s thyroiditis, hypothyroidism, graves’ orbitopathy, graves’ ophthalmopathy

## Abstract

Graves’ ophthalmopathy (GO) is commonly associated with hyperthyroidism secondary to Graves’ disease (GD). Although rare, there have been case reports of it occurring in patients who are hypothyroid with underlying Hashimoto’s thyroiditis (HT), as well as in euthyroid patients. Below, we describe a case of GO developing in a patient who has hypothyroidism secondary to HT successfully treated with high-dose steroids.

We present a case of a 53-year-old female known to have primary hypothyroidism (Hashimoto’s thyroiditis) diagnosed at the age of 39 years and has been on levothyroxine since diagnosis. She presented to our endocrine clinic complaining of new-onset diplopia and periorbital swelling for five months. There is no previous hyperthyroid state or radioactive iodine therapy. Examination showed left-sided upper and lower eyelid swelling, limited abduction with diplopia, and mild punctate keratopathy. Laboratory investigation revealed positive thyroid-stimulating immunoglobulin of 500 IU/mL (normal value: <140 IU/mL) with thyroid-stimulating hormone (TSH) and free thyroxine (FT4) in the euthyroid range. She was found to have a small heterogeneous thyroid gland on ultrasound suggestive of atrophic thyroiditis, and magnetic resonance imaging (MRI) of the orbits demonstrated bilateral ocular proptosis with extraocular muscle enlargement.

The patient was diagnosed with active moderate-severe isolated GO with a background of HT, clinically and biochemically euthyroid on levothyroxine. She was referred to an ophthalmologist and was started on a course of high-dose oral prednisone tapered over three months, which was followed by oral selenium and botox injections to both medial recti muscles.

Graves’ ophthalmopathy is an uncommon presentation in hypothyroid and euthyroid patients but should be considered in the differential diagnosis. The incidence varies between studies from 2% to 7.5%. Awareness of this clinical presentation is important, as early detection and treatment can prevent visual complications. To date, there are no clear guidelines on how to treat GO with underlying HT. Treating our patient with high-dose steroids extrapolated from treating GO secondary to GD showed significant improvement in her symptoms.

## Introduction

Graves’ ophthalmopathy (GO), also named Graves’ orbitopathy, thyroid-associated ophthalmopathy, or thyroid eye disease, is a potentially sight-threatening disease. It occurs in 25%-50% of patients with hyperthyroidism secondary to Graves’ disease (GD) [1]. There are only a few case reports describing it in patients who are euthyroid or hypothyroid secondary to Hashimoto’s thyroiditis (HT) [2,3].

Graves’ ophthalmopathy is an autoimmune disorder with genetic and environmental factors playing a role in its development [4]. Its pathogenesis is still not well understood; however, it is thought that the mechanism behind developing GO in hypothyroid or euthyroid patients is related to the presence of thyroid-stimulating hormone (TSH) receptor stimulating autoantibodies that target orbital fibroblasts causing inflammation and cellular infiltration of the surrounding tissue [5,6]. Similar to the effect of a hyperthyroid state, hypothyroidism can negatively impact the course and progression of GO, while restoration of euthyroidism is often associated with stabilization and improvement of GO [7,8]. Thereby, achieving and maintaining a euthyroid state is essential.

This case reports the development of GO in a patient who has hypothyroidism secondary to HT. She was treated with high-dose steroids, which showed significant improvement in her symptoms. The reporting of this case describes the diagnosis and management of a patient presenting with this rare entity.

This case report was previously presented as a meeting poster at the Ninth International Conference of Endocrinology and Diabetes (9th ICED) on February 19, 2022.

## Case presentation

We present a case of a 53-year-old female nonsmoker known to have primary hypothyroidism (Hashimoto’s thyroiditis) diagnosed at the age of 39 years and has been on levothyroxine since diagnosis. She presented to our endocrine clinic complaining of diplopia and periorbital swelling for the past five months. The diplopia occurs on the left gaze while both eyes are open, and it resolves with the closure of either eye. There was no history of ocular surgeries, injections, or trauma and no previous hyperthyroid state or radioactive iodine therapy.

On physical examination, there was no goiter, bruit, palpable lymphadenopathy, tremor, or pretibial myxedema. Ophthalmic examination showed left-sided upper and lower eyelid swelling, limited abduction with diplopia, and mild punctate keratopathy.

**Figure 1:**
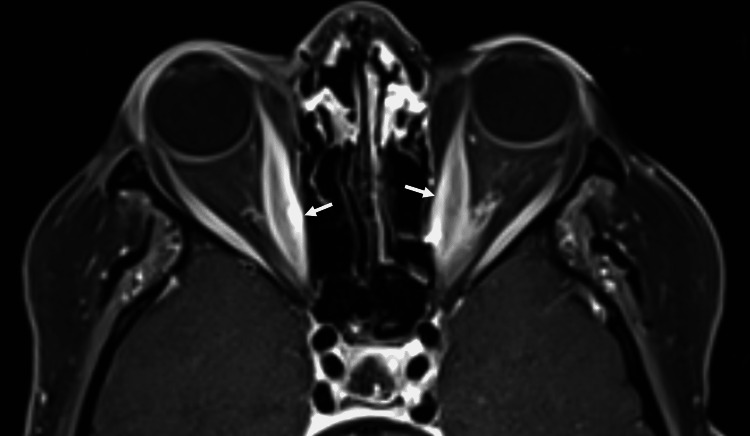
Contrast-enhanced magnetic resonance imaging (MRI) of the orbits showing bilateral ocular proptosis with medial recti muscle enlargement (white arrows).

Laboratory investigation revealed TSH of 3.6 mIU/L (normal range: 0.25-5 mIU/L), free thyroxine (FT4) of 14.6 pmol/L (normal range: 1.5-22.7 pmol/L), positive thyroid peroxidase antibody of 173.9 units (normal value: <100 units), and positive thyroid-stimulating immunoglobulin of 500 IU/mL (normal value: <140 IU/mL). On ultrasound, the thyroid gland was small and heterogeneous. Magnetic resonance imaging (MRI) of the orbits demonstrated bilateral ocular proptosis with extraocular muscle enlargement, particularly in the medial, inferior, superior rectus, and levator palpebrae superioris muscles (Figure 1).

Our impression was active moderate-severe isolated GO with a background of HT, clinically and biochemically euthyroid on levothyroxine. Both the endocrinologist and ophthalmologist were involved in the patient’s care. She was treated medically based on the activity and severity of her eye disease with high-dose oral prednisone, followed by oral selenium and extraocular muscle botox injections, extrapolated from the 2021 European Group on Graves’ Orbitopathy (EUGOGO) clinical practice guidelines for the medical management of Graves’ orbitopathy [9]. The patient reported a 50% improvement in her symptoms after the first month of therapy. During her last clinic visit, she had stable thyroid eye disease, and there was no need for further medical or surgical intervention.

## Discussion

Graves’ ophthalmopathy is a disease of the orbital tissue, usually occurring in association with autoimmune thyroid disease [10]. Although most cases are associated with a hyperthyroid state secondary to GD, there have been rare case reports of it occurring in patients who are hypothyroid with underlying HT [11]. The incidence of thyroid-associated ophthalmopathy in hypothyroid patients in the absence of a previous hyperthyroid state varied between studies from 2% to 7.5% [5,12].

The pathogenesis could be explained by the development of TSH receptor stimulating autoantibodies directed against the thyrotropin receptors expressed on orbital fibroblasts, causing cellular infiltration and inflammation of ocular muscles and the surrounding adipose tissue, resulting in their expansion and pressure buildup [13]. Clinically, there can be eye pain, redness, swelling, exophthalmos, visual impairment, and/or diplopia. Radiologic features on MRI include increased fat density, extraocular muscle enlargement, proptosis, eyelid edema, and enlarged lacrimal glands. Performing orbital imaging can help rule out other causes such as orbital mass lesions. The diagnosis of GO in the setting of HT can be made based on clinical and radiologic features and is supported by finding high titers of TSH receptor stimulating autoantibodies [11].

The patient’s hypothyroidism is most likely secondary to HT, as she had high titers of thyroid peroxidase antibodies and small heterogeneous thyroid on ultrasound. The recent development of eye symptoms with high titers of TSH receptor stimulating autoantibodies supports the diagnosis of new-onset GO.

It is important to be aware that some patients initially develop a hypothyroid state and then become hyperthyroid after many years, or vice versa. This spontaneous alternation in the thyroid state can be explained by the presence of two types of TSH-binding inhibitory immunoglobulins (TBII) either simultaneously or sequentially, which are the stimulatory and inhibitory TBII [14,15]. The thyroid state is dependent on the dominant antibody, which can alternate from one type to another even after many years. The dominance of the stimulatory TBII causes Graves’ hyperthyroidism, while the inhibitory TBII causes hypothyroidism. This has been demonstrated in a study evaluating the change in thyroid state in TBII-positive patients over 10 years. Two out of 34 hypothyroid patients with inhibitory TBII went on to develop hyperthyroidism with stimulatory TBII, and two out of 98 patients with Graves’ hyperthyroidism with stimulatory TBII developed hypothyroidism with positive inhibitory TBII [16].

To date, there are no clear guidelines on how to treat GO with underlying HT. There have been reports demonstrating improvement of the thyroid eye disease in hypothyroid and euthyroid patients when treated with high-dose intravenous (IV) steroids extrapolated from the treatment of patients with underlying GD [17]. There are other reports demonstrating clinical improvement in orbitopathy once the patient is initiated on levothyroxine and restored to a euthyroid state [18].

Treatment of GO with underlying GD based on EUGOGO guidelines depends on the activity and severity of the disease. Smoking cessation and achieving a euthyroid state are recommended for all patients. Local measures (ocular lubrication and botulinum toxin A injection) should be used when needed to achieve good symptomatic relief regardless of disease activity. Antioxidant therapy with selenium (in selenium-deficient areas) is recommended if the disease is active and mild. In the setting of active moderate-severe disease, high-dose steroids (IV preferred over oral) is the first line of therapy, either alone or in combination with other immunomodulatory therapy (mycophenolate, teprotumumab, rituximab, etc.). Sight-threatening disease should be treated with high-dose IV steroids, and if there is no response, then urgent orbital decompression surgery is required. When the disease is inactive, moderate-severe rehabilitative surgery (orbital decompression and extraocular muscle/eyelid procedures) can be considered [9].

## Conclusions

Graves’ ophthalmopathy in the setting of hypothyroidism is very rare; however, its incidence has been reported in adults with euthyroid and hypothyroid states. Awareness of the occurrence of GO in the setting of concurrent HT can aid in the early detection and treatment of this rare condition, which can help prevent sight-threatening complications. Patients with hypothyroidism and GO can be successfully treated with steroids, with the advancement to other immunomodulatory therapy if indicated, in a similar manner to patients with GO and hyperthyroidism. This requires the involvement of both the endocrinologist and ophthalmologist in the patient’s care.
